# Supplementary material for: Empirical methods for controlling false positives and estimating confidence in ChIP-Seq peaks
Source: BMC Bioinformatics. 2008 Dec 5;9:523. doi: 10.1186/1471-2105-9-523 (PMC2628906; doi:10.1186/1471-2105-9-523)
Supplement: Additional File 1 — A variety of html documents from the USeq web site detailing the available applications, their best usage, output file type descriptions, command line menus, etc. [file 1471-2105-9-523-S1.zip › USeqUserGuides/JavaDoc.zip!/JavaDoc/meme/MemeResults.html]

MemeResults


|  |  |  |
| --- | --- | --- |
| **Overview** \| **Package** \| **Class** \| **Use** \| **Tree** \| **Deprecated** \| **Index** \| **Help** | |  |
| **PREV CLASS**   **NEXT CLASS** | **FRAMES**    **NO FRAMES**     **All Classes** |  |
| SUMMARY: NESTED \| FIELD \| CONSTR \| METHOD | DETAIL: FIELD \| CONSTR \| METHOD |  |


---


## meme Class MemeResults

```
java.lang.Object
  meme.MemeResults
```

---

``` public class MemeResults extends java.lang.Object ```

Holds the results from various parts of MemeR.

---

**Constructor Summary**

`MemeResults()`


| **Method Summary** | |
| --- | --- |
| `void` | `appendMemeParserReport(java.lang.String data)` |
| `void` | `appendMemeReports(java.lang.String data)` |
| `void` | `appendMotifSearchResults(java.lang.String data)` |
| `void` | `appendSelexOligoFiles(java.lang.String data)` |
| `java.lang.String` | `getMemeReports()` |
| `java.lang.String` | `getMotifHitPlot()`             Rude and crude method to plot the percent hits of each motif to each file using data from motifHitSummary |
| `java.lang.String` | `getMotifHitPlotText()` |
| `java.lang.String` | `getMotifHitsSummary()`             Method to return a summary of all the motif hits to each file, modify this to build an HTML table |
| `java.lang.String` | `getMotifHitsSummaryText()` |
| `java.lang.String` | `getMotifSummary()`             Method to return a summary of all the motifs generated by MemeR, modify this to build an HTML table |
| `java.lang.String` | `getMotifSummaryText()` |
| `java.lang.String` | `getResults()` |
| `void` | `makeFileNameHash()` |
| `void` | `printSave(java.lang.String newString)` |
| `void` | `setFileBaseName(java.lang.String data)` |
| `void` | `setMemeCmdLnArgs(java.lang.String data)` |
| `void` | `setMemeParserArrayRef(MemeParser[] data)` |
| `void` | `setMotifHitPlotText(java.lang.String data)` |
| `void` | `setMotifHitsSummaryText(java.lang.String data)` |
| `void` | `setMotifResults(java.util.ArrayList data)` |
| `void` | `setMotifSummaryText(java.lang.String data)` |
| `void` | `setResultsDirectory(java.lang.String data)` |
| `void` | `sortMotifSearchResults()`             method to sort the array of MotifSearchResult objects, also sets two fields based on the this.fileNameHash |
| `java.lang.String` | `toString()` |

**Methods inherited from class java.lang.Object**

`equals, getClass, hashCode, notify, notifyAll, wait, wait, wait`

**Constructor Detail**

### MemeResults

```
public MemeResults()
```


**Method Detail**

### getMotifSummary

```
public java.lang.String getMotifSummary()
```

:   Method to return a summary of all the motifs generated by MemeR, modify this to build an HTML table

---


### sortMotifSearchResults

```
public void sortMotifSearchResults()
```

:   method to sort the array of MotifSearchResult objects, also sets two fields based on the this.fileNameHash

---


### getMotifHitsSummary

```
public java.lang.String getMotifHitsSummary()
```

:   Method to return a summary of all the motif hits to each file, modify this to build an HTML table

---


### getMotifHitPlot

```
public java.lang.String getMotifHitPlot()
```

:   Rude and crude method to plot the percent hits of each motif to each file using data from motifHitSummary

---


### printSave

```
public void printSave(java.lang.String newString)
```

---


### toString

```
public java.lang.String toString()
```

:   **Overrides:**: `toString` in class `java.lang.Object`

---


### makeFileNameHash

```
public void makeFileNameHash()
```

---


### getResults

```
public java.lang.String getResults()
```

---


### getMemeReports

```
public java.lang.String getMemeReports()
```

---


### getMotifSummaryText

```
public java.lang.String getMotifSummaryText()
```

---


### getMotifHitsSummaryText

```
public java.lang.String getMotifHitsSummaryText()
```

---


### getMotifHitPlotText

```
public java.lang.String getMotifHitPlotText()
```

---


### setMemeParserArrayRef

```
public void setMemeParserArrayRef(MemeParser[] data)
```

---


### setMotifHitPlotText

```
public void setMotifHitPlotText(java.lang.String data)
```

---


### setMotifSummaryText

```
public void setMotifSummaryText(java.lang.String data)
```

---


### setMotifHitsSummaryText

```
public void setMotifHitsSummaryText(java.lang.String data)
```

---


### appendSelexOligoFiles

```
public void appendSelexOligoFiles(java.lang.String data)
```

---


### setMemeCmdLnArgs

```
public void setMemeCmdLnArgs(java.lang.String data)
```

---


### setFileBaseName

```
public void setFileBaseName(java.lang.String data)
```

---


### setResultsDirectory

```
public void setResultsDirectory(java.lang.String data)
```

---


### appendMemeParserReport

```
public void appendMemeParserReport(java.lang.String data)
```

---


### appendMemeReports

```
public void appendMemeReports(java.lang.String data)
```

---


### appendMotifSearchResults

```
public void appendMotifSearchResults(java.lang.String data)
```

---


### setMotifResults

```
public void setMotifResults(java.util.ArrayList data)
```


---


|  |  |  |
| --- | --- | --- |
| **Overview** \| **Package** \| **Class** \| **Use** \| **Tree** \| **Deprecated** \| **Index** \| **Help** | |  |
| **PREV CLASS**   **NEXT CLASS** | **FRAMES**    **NO FRAMES**     **All Classes** |  |
| SUMMARY: NESTED \| FIELD \| CONSTR \| METHOD | DETAIL: FIELD \| CONSTR \| METHOD |  |


---
